# A cost-minimization analysis of anti-VEGFs for the treatment of neovascular age-related macular degeneration in the Netherlands

**DOI:** 10.1007/s00417-024-06588-6

**Published:** 2024-09-25

**Authors:** Sara W. Quist, Hidde Nab, Maarten Postma, Sankha Amarakoon, Freekje van Asten, Roel Freriks

**Affiliations:** 1 Department of Health Sciences, University Medical Center Groningen, Groningen, The Netherlands; 2 Asc Academics, Groningen, The Netherlands; 3 Department of Economics, Econometrics and Finance, University of Groningen, Groningen, The Netherlands; 4 University Eye Clinic Maastricht, MUMC+, Maastricht, The Netherlands; 5 Department of Health Technology and Services Research, TechMed Centre, University of Twente, Enschede, Netherlands

**Keywords:** Anti-VEGFs, Neovascular age-related macular degeneration, Cost-minimisation

## Abstract

**Objective:**

Age-related macular degeneration (AMD) is the main cause of severe vision loss globally. Neovascular AMD (nAMD) is an advanced stage of AMD treated with anti-vascular endothelial growth factors (anti-VEGFs). Although anti-VEGF treatment is effective, the frequent intravitreal injections place a burden on patients, (in)formal caregivers, and clinics. This study assesses the health-economic impact of anti-VEGF agents with lower injection frequency that have the potential to reduce treatment burden and compares it to the standard of care.

**Methods:**

We developed a cost-minimization model to evaluate the direct medical costs associated with first-line unilateral anti-VEGF treatment across a 3-year time horizon in the Netherlands. The analysis compared aflibercept 8 mg, aflibercept 2 mg, bevacizumab, faricimab, and ranibizumab. Our model adopted a treat-and-extend (T&E) regimen for aflibercept 2 mg, bevacizumab, and ranibizumab. For aflibercept 8 mg, a flexible regimen that was extendable up to 24 weeks was applied, while faricimab followed a flexible regimen that was extendable up to 16 weeks. Additionally, since list prices may vary from net prices, we calculated the break-even price for each anti-VEGF in comparison to bevacizumab, which is the recommended first-line treatment due to its low medication price.

**Results:**

Based on list prices, aflibercept 8 mg led to the lowest treatment costs (€16,251 per patient over a 3-year time horizon), closely followed by bevacizumab (€17,616 per patient over a 3-year time horizon). Ranibizumab led to the highest per-patient costs (€31,746 over a 3-year time horizon). For bevacizumab, most costs were attributable to administration, while for the other anti-VEGFs, most were attributable to medication. Aflibercept 8 mg is cost-saving compared to bevacizumab at their medication prices at the time of writing. Aflibercept 2 mg, faricimab, and ranibizumab should be priced below €488, €591, and €75, respectively. To be cost-equal to bevacizumab with current list prices, anti-VEGFs should be administered with a maximum of 12.7 to 13.8 injections over a 3-year time horizon.

**Conclusion:**

According to the injection frequency observed in clinical trials, aflibercept 8 mg would be the anti-VEGF that generates the lowest per-patient healthcare costs for the treatment of nAMD in the Netherlands after a treatment period of three years. Our study indicates that anti-VEGF drugs with a lower injection frequency might provide a cost-saving solution to the increasing burden of anti-VEGF treatment on the healthcare system.
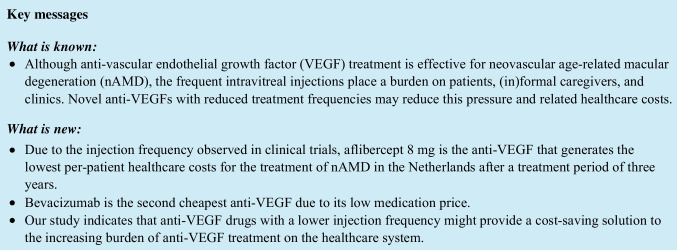

## Introduction

In 2021, there were more than 146,000 patients with age-related macular degeneration (AMD) in the Netherlands, and this number increases by 13,700 patients annually, exacerbated by the ageing of the population [1, 2]. Approximately 10%–15% of AMD patients develop the advanced stage neovascular age-related macular degeneration (nAMD) [3]. Due to its rapid growth, the number of European patients with advanced AMD is likely to increase from 2.7 million in 2013 to 3.6 or 4.8 million by 2040 [2]. AMD and nAMD are the main causes of vision loss worldwide [3] that, due to their negative impact on general and mental health, significantly affect patients’ quality of life [4–6].

Currently, the first-line treatment for patients with nAMD entails anti-vascular endothelial growth factors (anti-VEGFs) administered through intravitreal injections [7]. The anti-VEGFs that are presently available in the Netherlands include aflibercept 2 mg, bevacizumab, brolucizumab, faricimab, ranibizumab, and soon aflibercept 8 mg [7]. Guidelines recommend initiating nAMD treatment with bevacizumab due to its lower medication price and efficiency comparable to other anti-VEGFs. In cases of insufficient treatment response, bevacizumab may be switched to aflibercept 2 mg or ranibizumab [7]. Brolucizumab is recommended as a last option as it increases the risk of intraocular inflammation post-injection in patients [7]. Faricimab and aflibercept 8 mg obtained European marketing authorizations in 2022 and 2024, respectively [8, 9]. Although all previously mentioned anti-VEGFs are believed to induce a similar vision gain, the number of injections needed to reach and maintain this effect varies between anti-VEGFs and the type of regimen [10, 11].

A reduced injection frequency may benefit patients, (in)formal caregivers, and clinics as it decreases the burden associated with anti-VEGF treatment. The frequent intravitreal injections and check-ups that anti-VEGF treatment require are time-consuming [7]. Accordingly, studies show that patients prioritize effectiveness in their treatment, but given the same effect, they prefer treatment regimens that require fewer hospital visits [12]. In addition, intravitreal injections induce anxiety by some patients, especially those who recently started the treatment [13, 14]. At the same time, Dutch ophthalmic healthcare is under pressure; ophthalmology has the third longest waiting list of all hospital specializations in the Netherlands (i.e., 8.1 weeks on average), and the annual medical costs of eye-related disorders amount to approximately one billion euros [15, 16].

Over the years, several anti-VEGF treatment strategies have been introduced to decrease the number of injections while maintaining similar vision gains. Compared to older fixed regimes, the treat-and-extend (T&E) regimen has been shown to reduce both over- and undertreatment by adjusting the treatment intervals to the patient’s disease activity and is therefore recommended as a treatment regimen for nAMD in the Dutch guideline [7, 17]. In the T&E regimen, clinical trials showed that the treatment intervals of bevacizumab and ranibizumab could be extended up to 12-weeks and the treatment intervals of aflibercept 2 mg and faricimab could be extended up to 16 weeks [18–20]. Moreover, the PULSAR trial showed that the recently introduced aflibercept 8 mg could even exceed the maximum 16-week intervals recommended by Dutch guidelines [7, 11]. At week 96, 78% of patients randomized to aflibercept 8 mg exceeded a flexible 16-week interval, and 53% of them exceeded a 20-week interval [21, 22].

Since anti-VEGFs have comparable effects, treatment costs should be an important factor when selecting an anti-VEGF to help reduce the (financial) strain on ophthalmic healthcare. The use of a broad healthcare perspective instead of solely direct medication costs can contribute to an appropriate decision. Currently, the Dutch treatment guideline recommends a T&E regimen with bevacizumab as first-line treatment, primarily due to its calculated budget impact and previous cost-effectiveness analyses [7]. This budget-impact calculation is limited to medication costs and assumes that efficacy, durability, and treatment exposure among various anti-VEGFs are similar [7]. Conversely, a prior cost-minimization analysis calculated the per-patient costs of anti-VEGF treatment using clinical trial data and encompassed all healthcare-related costs [23]. Although this analysis also indicated that bevacizumab was linked to the lowest total healthcare costs [23], the disparity in per-patient costs, when compared to other anti-VEGFs, was less notable than the difference identified in the budget-impact analysis carried out for the Dutch treatment guidelines [7]. This outcome was mainly caused by the higher number of administrations and screenings in a T&E regimen for bevacizumab compared to the other anti-VEGFs.

Although informative, neither the previous cost-minimization analysis nor the budget-impact analysis of the Dutch treatment guideline included the novel aflibercept 8 mg and faricimab [7, 23]. Therefore, this study aims to compare the direct medical costs of the novel anti-VEGFs (aflibercept 8 mg and faricimab) to the current standard of care (bevacizumab, aflibercept 2 mg, and ranibizumab) for unilateral treatment of treatment-naïve nAMD patients in the Netherlands.

## Methods

Our study is a follow-up of the previous cost-minimization analysis that estimated all direct medical costs relevant to one-eye anti-VEGF treatment in treatment-naïve nAMD patients [23]. The cost-minimization model was developed in Microsoft Excel (Redmond, WA, USA) and used a 3-year time horizon as Dutch guidelines prescribe this period as the minimum time to estimate the impact of treatment on the healthcare payer [24]. Given the chronic nature of nAMD [24], a shorter time frame would inadequately reflect the costs associated with anti-VEGF treatment. However, as clinical trials lasted two years, a longer time horizon would lead to too much uncertainty in extrapolation. Our analysis compares aflibercept 2 mg, aflibercept 8 mg, bevacizumab, faricimab, and ranibizumab and includes optimal clinical trial regimens, specifically those that minimize injection frequency without compromising vision (gain) maintenance for all anti-VEGFs currently available in the Netherlands. The analysis did not include brolucizumab, as this anti-VEGF treatment is generally not used in naïve patients due to its elevated risk for intraocular inflammation [7]. As the clinical trials demonstrated the non-inferiority of anti-VEGFs in vision gains from baseline, the model omitted the treatment effect [18, 20, 22, 25–27]. The analysis was conducted from a healthcare perspective to inform medical stakeholders (i.e., doctors, healthcare insurance companies, healthcare policy makers, and pharmacists) of the impact of anti-VEGF treatment and their regimens. The most important study characteristics are summarized in Table 1, and study assumptions are presented in Appendix 1 Table 8.

**Table 1:** Overview of model characteristics

	Input
Model type	Cost-minimization analysis
Perspective	Healthcare payer
Time horizon	3-years
Included anti-VEGFs and their regimens	Aflibercept 2 mg (T&E with an injection interval of up to 16 weeks) Aflibercept 8 mg (Q16 arm, with an injection interval of up to 24 weeks) Bevacizumab (T&E with an injection interval of up to 12 weeks) Faricimab (Flexible with an injection interval of up to 16 weeks) Ranibizumab (T&E with an injection interval of up to 12 weeks)
Cost parameters	Medication Administration Clinic visits Monitoring visits Adverse events
Outcomes	Costs per patient (based on unilateral treatment) Break-even price relative to cheapest anti-VEGF subjective to injection frequency OWSA

*OWSA* One-way sensitivity analysis; *T&E* Treat-and-extend

### Included regimens

Our study compared anti-VEGFs in their optimal clinical trial regimens (i.e., regimens that minimise injection frequency without compromising vision gain maintenance) which include the T&E regimen for the established anti-VEGFs and a flexible up to 24 weeks regimen for aflibercept 8 mg and flexible up to 16-week regimen for faricimab.

The T&E regimen is recommended by the Dutch guideline for AMD and by experts because it has proven effective in preventing both over- and undertreatment [7, 17]. As seen in most clinical studies, a T&E regimen consists of a loading phase with three monthly injections, followed by a flexible treatment interval that ranges from 4 to 16 weeks, depending on disease activity [17, 20, 28, 29]. The disease activity is assessed during a monitoring visit that preferably coincides with the injection visit. Dutch guidelines indicate that patients should be diagnosed with spectral domain optical coherence tomography (SD-OCT), vision assessment, and fundoscopy to determine the presence of intraretinal and/or subretinal fluid, vision loss, and haemorrhage (Appendix 2 Table 9) [7]. A similar assessment should be repeated at the end of the loading phase [7]. In the subsequent assessments, disease activity should be evaluated with an SD-OCT to establish the presence of intraretinal and/or subretinal fluid [7]. Table 2 summarizes the T&E regimens that were studied in the included clinical trials of the established anti-VEGFs.

**Table 2:** Overview of T&E regimen characteristics of anti-VEGFs based on clinical trials indicated by their names

	Treatment schedule				Treat-and-extend criteria		
Treatment (regimen)	Loading phase	Min. -max. injection interval	Interval adjustments	Patient distribution at end year 2	Shorten	Maintain	Extend
Aflibercept 2 mg Based on ALTAIR (96 weeks) and ARIES (104 weeks) [early T&E] [20, 30]	3 monthly doses	8 to 16 weeks	ALTAIR: 2 weeks or 4 weeks ARIES: 2 weeks, but 4 weeks when the eye was completely dry	ALTAIR ≥ 12 weeks: 56.9% ≥ 16 weeks: 41.5% ARIES: < 8 weeks: 5.7% 8 weeks: 27.4% 10 weeks: 19.8% 12 weeks: 8.5% 14 weeks: 8.5% ≥ 16 weeks: 30.2%	ALTAIR: New or persistent (intra- or subretinal) fluid with unchanged or increased fluid volume compared to previous visit or recurrent fluid Loss of BCVA of ≥ 5 letters compared in conjunction with recurrent fluid to the previous visit An increase in CRT of ≥ 100 µm t the central 1 mm compared with the lowest previous value New-onset neovascularization New macular haemorrhage ARIES: Extending criteria were not met	ALTAIR: If none of the criteria for shortening were met and residual fluid had decreased from the previous visit	ALTAIR: If none of the criteria for shortening were met, and there was no fluid on OCT, then the interval was extended ARIES: The absence of intraretinal fluid, absence of new neovascularization or hemorrhage, and subretinal fluid not exceeding 50 µm in thickness
Bevacizumab Based on LUCAS (104 weeks)[18]	Monthly doses until no disease activities	4 to 12 weeks	2-weeks	4 weeks: 46.7% 6 weeks: 12.0% 8 weeks: 9.0% 10 weeks: 7.2% 12 weeks: 25.1%	LUCAS: Any signs of fluid on OCT, new or persistent haemorrhage or dye leakage, or increased lesion size on FA		LUCAS: No signs of recurrent disease
Ranibizumab Based on TREX (104 weeks), TREND (52 weeks), LUCAS (104 weeks), CANTREAT (104 weeks), InEye (52 weeks) [18, 28, 29, 31, 32]	3 monthly doses	4 to 12 weeks	2-weeks	LUCAS: 4 weeks: 32.9% 6 weeks: 14.7% 8 weeks: 4.7% 10 weeks: 10.6% 12 weeks: 37.1% TREX: ≥ 8 weeks: 47% 11–12 weeks: 37% CANTREAT ≥ 8 weeks: 73.7% 12 weeks: 43.1%	LUCAS: Any signs of fluid on OCT, new or persistent haemorrhage or dye leakage, or increased lesion size on FA TREX: Presence of intraretinal and subretinal fluid TREND: any fluid and unspecified vision loss CANTREAT: The presence of any fluid, vision loss of more than 5 ETDRS letters, presence of new haemorrhage or progression of choroidal neovascularization, or a combination thereof InEye: VA (loss of ≥ 5 letters) and/or one of the following criteria: new haemorrhages on fundus examination, persistent or recurrent intraretinal or subretinal fluid on spectral-domain OCT (SD-OCT), and leakage from CNV on FA (FA was only mandatory per protocol (PP) at the beginning and at the end of the study)		LUCAS: No signs of recurrent disease TREX: Resolution of intraretinal and subretinal fluid TREND: no fluid and no vision loss CANTREAT: Gain in VA of 3 ETDRS letters or more (or no loss of more than 5 letters) from the prior month; no clinical evidence of lesion growth, fluid, or blood; and no intraretinal or subretinal fluid seen on OCT InEye: No disease activity

*BCVA* Best-corrected visual acuity; *CNV* Choroidal neovascularization; *CRT* Central retinal thickness; *T&E* Treat-and-extend; *SD-OCT* Spectral domain optical coherence tomography

Currently, the summary of product characteristics (SmPC) of faricimab recommends the flexible 16-week regimen that was studied in the TENAYA and LUCERNE trials [8]. The flexible regimen for faricimab starts with a loading phase of one injection per month for four consecutive doses and is subsequently followed by an injection every eight weeks, every 12 weeks, or every 16 weeks, depending on disease activity at week 20 and/or 24. For aflibercept 8 mg, its SmPC recommends a flexible regimen of up to 20 or 24 weeks [9]. To model this regimen, we used the randomized Q16 arm from the PULSAR trial. The flexible up to 24 weeks regimen starts with a loading dose of one injection per month for three consecutive doses, followed by an extension of injection intervals up to four and subsequently up to five and six months (20 and 24 weeks) based on visual and/or anatomic outcomes. Table 3 presents the regimen characteristics of the novel anti-VEGFs.

**Table 3:** Overview of characteristics of anti-VEGF regimens that are not T&E based on clinical trials indicated by their names

	Anti-VEGFs with another regimen				
	Treatment schedule				Regimen modification Description of regimen modification criteria
Treatment (regimen)	Loading phase	Minimum and maximum interval	Patient distribution at end year 2	Schedule description	
Aflibercept 8 mg (Q16 arm) Based on PULSAR (96 weeks) [11, 22]	Yes (3 monthly doses)	8 to 24 weeks	≥ 12 weeks: 89% ≥ 16 weeks: 83% 20 weeks: 16% 24 weeks: 27%	Start 16-week injection interval regimen adjusted to an 8-week injection interval if criteria for shortening injection interval were met at week 16 or 20 and to a 12-week injection interval weekly if criteria were met at week 24 First year No injection interval extension is allowed in the first year; only shortening. The minimum injection interval was 8 weeks. Shortening by 4-week increments from week 24 onwards when criteria were met Second year Shortening injection interval by 4-week increments when criteria were met (minimum interval 8 weeks) Extension of injection interval by 4-week increments from week 52 onwards with a maximum of 24 weeks when criteria were met	Criteria for interval shortening (first and second year): > 5-letter loss in BCVA compared with week 12 due to persistent or worsening nAMD, AND > 25 µm increase in CST compared with week 12 OR foveal haemorrhage OR new-onset foveal neovascularization Criteria for interval extension (second year): < 5-letter loss in BCVA compared with week 12, AND No fluid at the central subfield on OCT, AND No new onset foveal haemorrhage or foveal neovascularization
Faricimab (flexible regimen) Based on TENAYA and LUCERNCE (104 weeks) [27]	Yes (4 monthly doses)	8 to 16 weeks	TENAYA 8 weeks: 25.8% 12 weeks: 15.1% 16 weeks: 59.0% LUCERNE 8 weeks: 18.8% 12 weeks: 14.3% 16 weeks: 66.9%	First year 8-week injection interval if criteria for shortening injection interval were met at week 20 and 12-week injection interval if criteria were met at week 24. 16-week injection interval if no criteria were met in weeks 20 and 24 Second year Personalized treatment interval from week 60 onwards that is adjusted with 4-week increments for extension and 4 to 8- weeks for reduction (minimum interval 8 weeks)	Criteria at weeks 20 and 24 An increase of > 50 µm in by SD-OCT measured CST compared with the average CST value in the two previous scheduled visits, OR An increase of ≥ 75 µm CST compared with the lowest CST value with the lowest value in the two previous scheduled visits, OR A decrease of ≥ 5 letters in BCVA compared with the average BCVA value over the previous two scheduled visits, OR A decrease of ≥ 10 letters in BCVA compared with the highest BCVA value recorded at either of the previous two scheduled visits, owing to nAMD disease activity OR Presence of new macular haemorrhage, owing to nAMD disease activity OR Presence of new macular haemorrhage Week 24 Significant nAMD activity that does not meet criteria but requires immediate treatment

*BCVA* Best-corrected visual acuity; *CNV* Choroidal neovascularization; *CRT* Central retinal thickness; *CST* Central subfield thickness; *ETDRS* Early Treatment Diabetic Retinopathy Study; *SD-OCT* Spectral domain optical coherence tomography

### Injection and monitoring frequency

The injection frequency of each anti-VEGF in every treatment regimen was retrieved from Phase 3 or 4 prospective randomized clinical trials that measured change in visual acuity as the primary outcome in patients with unilateral treatment-naïve nAMD (Table 4, Appendix 3 Table 10). Trials that had a 96-week duration were extrapolated to 104 weeks. In addition, we assumed that the number of injections in year 3 was equal to year 2, based on real-world evidence [33–37]. When multiple trials were available, the injection frequency was determined by calculating the weighted average based on the trial population. During the loading phase, two monitoring visits were assumed: one at treatment initiation and one during the last administration. Subsequently, a monitoring visit was assumed for every administration, regardless of the discrepancy in clinical trial regimens.

**Table 4:** Overview of included injection and monitoring frequency based on the weighted average of clinical trials

	The mean number of injections			Number of monitoring visits			Sources
	Year 1	Year 2	Year 3	Year 1	Year 2	Year 3	
Aflibercept 2 mg	7.2	4.3	4.3	6.2	4.3	4.3	A weighted average of ALTAIR (T&E with 2-week adjustments), ARIES (early T&E) [20, 30]
Aflibercept 8 mg	5.4	3.3	3.3	4.4	3.3	3.3	PULSAR (8q16 arm) [11, 21, 22]
Bevacizumab	9.0	9.2	9.2	8.0	9.2	9.2	LUCAS [18]
Faricimab	6.8	3.8	3.8	5.8	3.8	3.8	TENAYA, LUCERNE [26, 27]
Ranibizumab	8.8	8.2	8.2	7.8	8.2	8.2	A weighted average of TREX, TREND, LUCAS, CANTREAT, InEye [18, 28, 29, 31, 32]

### Resource use and costs

All direct healthcare costs identified in literature relevant to medication, administration, hospital visits, imaging, and adverse events (AEs) were considered (Table 5). They were based on the Dutch literature and indexed to November 2023 using consumer price indices from Statistics Netherlands [38, 39]. Since bevacizumab is used off-label and is often prepared in hospital pharmacies, its medication price was calculated based on the time and materials that were used by medical staff to prepare it, while the medication prices of all other anti-VEGFs were based on the Dutch list prices (i.e., Z-index) [23, 40]. For each administration, the costs included medication, hospital visits, and intravitreal injections [38, 41, 42]. Each monitoring visit included imaging costs [7, 38, 41]. In each regimen, the first and second monitoring visits consisted of an SD-OCT, vision assessment, and fundus photography, while the subsequent monitoring visits consisted of solely an SD-OCT [7]. We assumed that all monitoring visits were performed at the same time as the administration of the intravitreal injection. Only AEs related to the administration of an intravitreal injection were implemented because an equal effect of each anti-VEGF was assumed. This assumption is substantiated by the comparable occurrence of AEs that were found in non-inferiority clinical trials [10, 25]. The assumed risk per injection was based on the study by Elshout et al. [38].

**Table 5:** Overview of included health care resources, costs and frequencies of use

	Cost per implementation	Implementation frequency	Source
Medication			
Aflibercept 2 mg	€724	Every administration	Z-index (list price)
Aflibercept 8 mg	€724	Every administration	Z-index (list price)
Bevacizumab	€18	Every administration	Calculation based on pharmacy preparation [23]
Faricimab	€730	Every administration	Z-index (list price)
Ranibizumab	€637	Every administration	Z-index (list price)
Administration			
Intravitreal injection	€475	Every administration	DBC: 079799020 Ophthalmology Society guideline [7]
Monitoring visits and imaging			
Clinical visits	€90	Every monitoring visit	DBC: 079699010
SD-OCT	€51	Every injection visit after the loading phase	Elshout et al. Dutch Ophthalmology Society guideline [7, 38]
SD-OCT, vision assessment, and fundoscopy	€180	Diagnosis at the start and end of the loading phase	DBC:79,699,011 Dutch Ophthalmology Society guideline [7]
Adverse events			
Endophthalmitis	€4,337	0.0004 per injection	Elshout et al. [38]
Retinal detachment	€2,819	0.0001 per injection	Elshout et al. [38]
Lens injury	€2,134	0.0001 per injection	Elshout et al. [38]
Intraocular haemorrhage	€281	0.0003 per injection	Elshout et al. [38]

*SD-OCT* Spectral domain optical coherence tomography

### Break-even analysis

In addition to the calculated per-patient costs for one-eye treatment, our model estimated the break-even price for each anti-VEGF relative to bevacizumab, which is the current first-line treatment in the Netherlands based on its relatively low medication price. The break-even price is the medication price of other anti-VEGFs, at which point the cost of treatment will equal that of bevacizumab. The break-even analysis aimed to improve accuracy, as injection frequencies are currently decreasing, and purchasing prices may differ from the list prices utilized in our study, especially in situations with competition or the availability of biosimilars. The break-even price was determined by calculating the direct healthcare costs without medication and by dividing this total by the number of injections per anti-VEGF. Additionally, to address uncertainty in injection frequency, we calculated a break-even price for ascending injection frequencies, which resulted in the break-even price per total number of injections over a 3-year time horizon.

### Sensitivity and scenario analyses

A one-way sensitivity analysis (OWSA) was performed to assess the model’s robustness. In the OWSA, all inputs were varied by ± 25% to assess the model’s sensitivity to input parameters. Additionally, we also performed a scenario analysis. In the first scenario, the medication price of bevacizumab was based on injections that were fully prepared by production pharmacies instead of hospital pharmacies (i.e., €39) [40, 42]. Given the 2-year duration of clinical trials, the implementation of a 3-year time horizon led to assumptions regarding the injection- and monitoring frequency and, therefore, uncertainty in the model. To account for this, we assessed the impact of a 2-year time horizon in the second scenario. On the other hand, 3-years is the minimum time horizon that is relevant to for payers. To assess the impact of extended anti-VEGF use, we also applied a 5-year time horizon, in which the injection frequency in the subsequent years was equal to year 2.

## Results

### Costs per patient

**Fig. 1:**
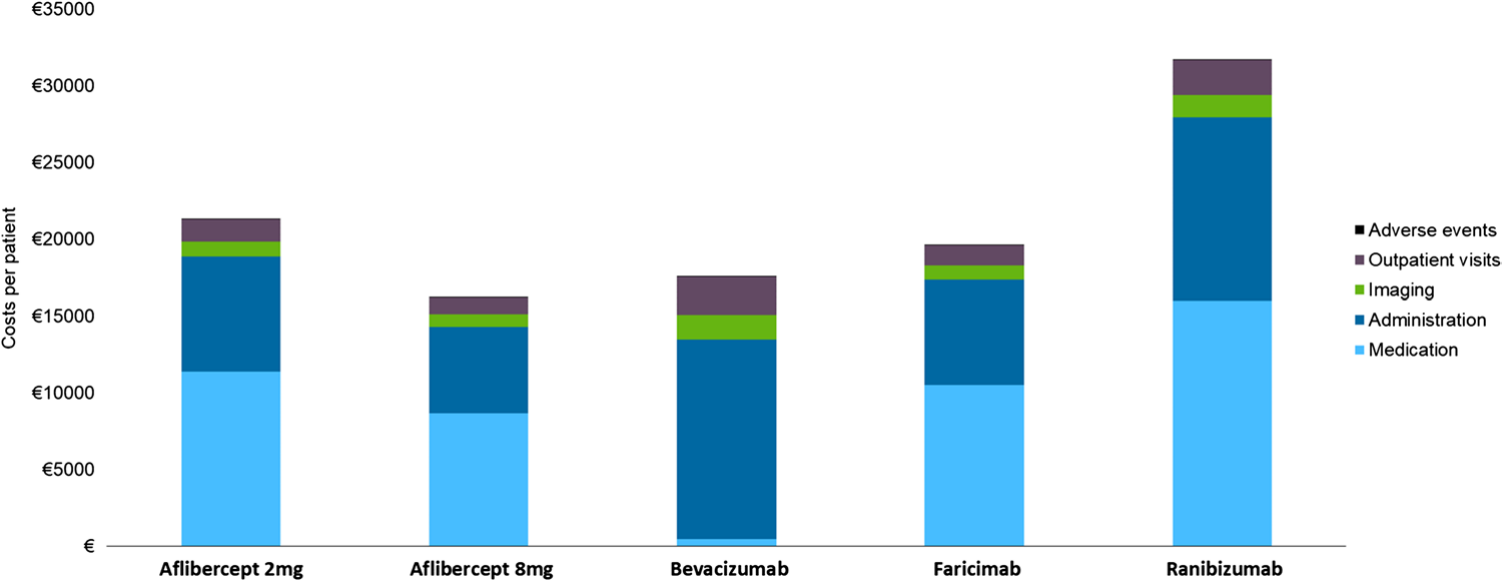
Costs per patient over a 3 year-time horizon categorized by medication, administration, imaging, outpatient visits, and AEs. Abbreviations: AE, adverse event

With €16,251 per patient over a 3-year time horizon, aflibercept 8 mg is associated with the lowest per-patient costs of all included anti-VEGFs (Table 6, Fig. 1). Bevacizumab is the second least expensive treatment and costs €17,616 per patient over a 3-year time horizon, which increases costs per patient by €1,365, compared to aflibercept 8 mg. Ranibizumab is the most expensive treatment option and costs €31,746 per patient over a 3-year time horizon. Medication is the main driver of the costs for all anti-VEGFs except for bevacizumab; it accounts for only 3% of the total costs of bevacizumab. Administration costs are the main cost driver of bevacizumab (i.e., 74%), due to its relatively high injection frequency. Additionally, to inform payers, Table 6 displays the costs in the initial treatment year and subsequent treatment years. All anti-VEGFs are more expensive in the first treatment year, with a relatively minor difference between the first and subsequent years for bevacizumab and ranibizumab.

**Table 6:** Medication, administration, imaging, outpatient visit, and total costs per patient over a 3-year time horizon and corresponding break-even price relative to bevacizumab

	Medication	Administration	Imaging	Outpatient visits	Adverse events	Total	Break-even price vs bevacizumab
Aflibercept 2 mg (Total)	€11,387 (53%)	€7,474 (35%)	€1,004 (5%)	€1,416 (7%)	€36 (0.2%)	€21,317	€488 (-33%)
Year 1	€5,177	€3,398	€570	€644	€17	€9,806	
Year 2 +	€3,105	€2,038	€217	€386	€10	€5,746	
Aflibercept 8 mg (Total)	€8,654 (53%)	€5,680 (35%)	€813 (5%)	€1,076 (7%)	€28 (0.2%)	€16,251	N/A
Year 1	€3,940	€2,586	€484	€490	€13	€7,513	
Year 2 +	€2,357	€1,547	€165	€293	€8	€4,369	
Bevacizumab (Total)	€477 (3%)	€13,015 (74%)	€1,594 (9%)	€2,466 (14%)	€64 (0.4%)	€17,616	Reference
Year 1	€157	€4,725	€664	€810	€21	€5,926	
Year 2 +	€160	€4,370	€465	€828	€21	€5,845	
Faricimab (Total)	€10,512 (54%)	€6,840 (35%)	€937 (5%)	€1,296 (7%)	€33 (0.2%)	€19,618	€591 (-19%)
Year 1	€4,964	€3,230	€552	€612	€16	€9,374	
Year 2 +	€2,774	€1,805	€192	€342	€9	€5,112	
Ranibizumab (Total)	€16,009 (50%)	€11,938 (38%)	€1,480 (5%)	€2,262 (7%)	€58 (0.2%)	€31,746	€75 (-88%)
Year 1	€5,626	€4,195	€655	€795	€20	€11,921	
Year 2 +	€5,192	€3,871	€412	€734	€19	€10,288

### Break-even price and analysis

**Fig. 2:**
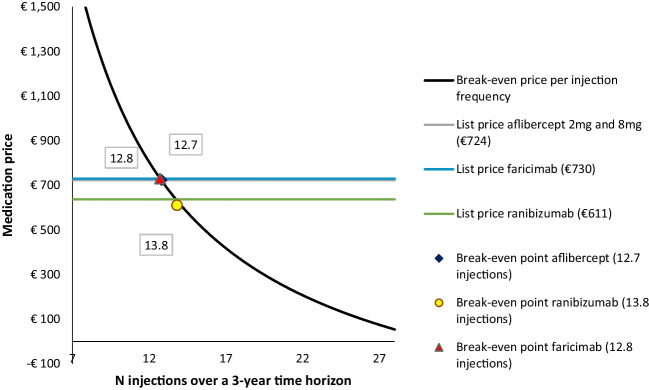
Break-even medication price per injection frequency over a 3-year time horizon, relative to bevacizumab

The break-even prices of the treatments were calculated in relation to bevacizumab, which is recommended as a first-line treatment in the Netherlands due to its low medication costs (Table 6) [7]. As aflibercept 8 mg was cost-saving when compared to bevacizumab at its list price at the time of writing, no break-even price was calculated. To be cost-equal to bevacizumab, the break-even prices of aflibercept 2 mg and faricimab are €488 (i.e., -33%) and €591 (-i.e.,19%), respectively. Ranibizumab needs the largest reduction in list price as its breakeven price is €75 (i.e., -88%). Additionally, Fig. 2 explores the break-even price of anti-VEGFs relative to their injection frequency over a 3-year time horizon. With the current list prices, to make the per-patient costs of anti-VEGFs equal to those of bevacizumab, the total number of injections required in a 3-year time horizon should range between 12.7 and 13.8. In comparison, bevacizumab requires 27 injections in this time frame.

### One-way sensitivity analysis

**Fig. 3:**
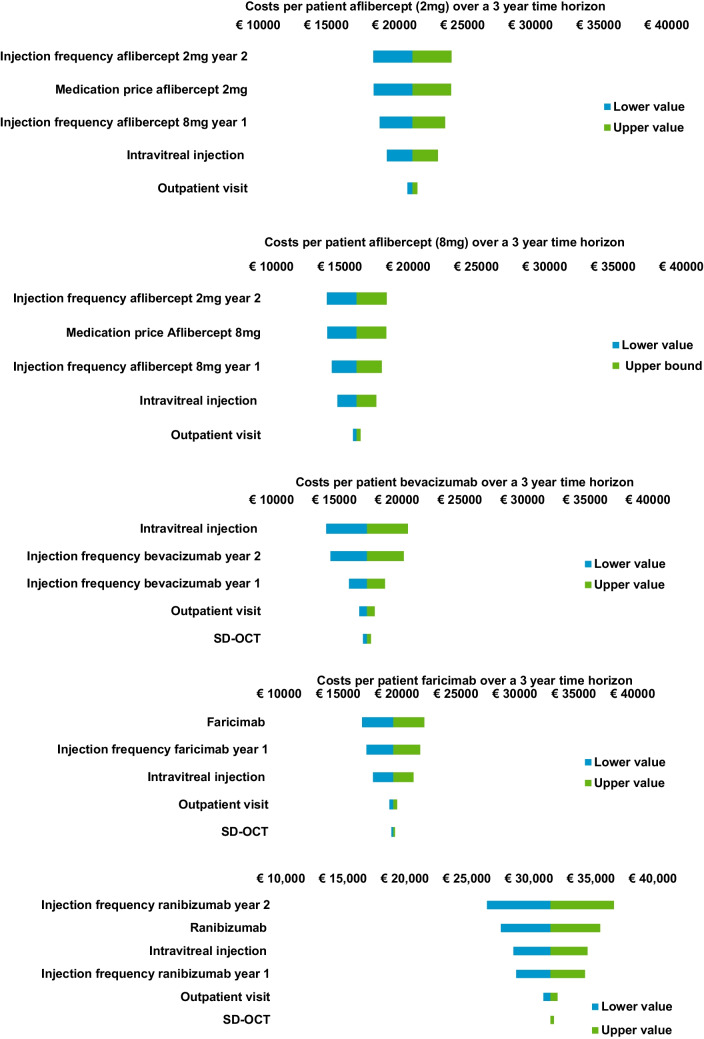
Tornado diagrams presenting outcomes of one-way sensitivity analysis

In the OWSA, all parameters were varied with a ± 25% interval (Appendix 4 Fig. 3). For all anti-VEGFs except bevacizumab, variation in medication price had the largest impact on the results. Other parameters with considerable impact on the total costs of all anti-VEGFs are injection frequency and the costs of intravitreal injections. For bevacizumab, intravitreal injection administration expenses were the largest impact on total costs.

### Scenario analysis

The scenario analysis assessed the impact of the use of bevacizumab injections that were fully prepared by production pharmacies, a 2-year time horizon, and a 5-year time horizon (Table 7). Due to the elevated medication prices, the use of fully prepared bevacizumab injections increases the total costs by €598, resulting in per-patient costs of €18,214 and slightly higher break-even prices for the other anti-VEGFs. When a 2-year time horizon is implemented, bevacizumab becomes cheaper than aflibercept 8 mg (i.e., €11,771 versus €11,882, respectively). With a reduction in the medication price to €710 (2%), aflibercept 8 mg is as costly as bevacizumab over a 2-year time horizon. A 5-year time horizon increases the difference in costs-savings of aflibercept 8 mg versus bevacizumab to €4,316. Their respective total costs amount to €24,989 and €29,305.

**Table 7:** Outcomes of the scenario analysis

Scenario 1: bevacizumab injections prepared by production pharmacies		
	Total	Break-even price vs bevacizumab
Aflibercept 8 mg	€16,251	N/A
Aflibercept 2 mg	€21,317	€526 (-27%)
Bevacizumab	€18,214	Reference
Faricimab	€20,034	€632 (-13%)
Ranibizumab	€31,746	€99 (-85%)
Scenario 2: use of a 2-year time horizon		
	Total	Break-even price vs bevacizumab
Aflibercept 8 mg	€11,882	€710 (-2%)
Aflibercept 2 mg	€15,562	€392 (-46%)
Bevacizumab	€11,771	Reference
Faricimab	€14,496	€473 (-35%)
Ranibizumab	€21,519	€63 (-90%)
Scenario3: use of 5-year time horizon		
	Total	Break-even price vs bevacizumab
Aflibercept 8 mg	€24,989	N/A
Aflibercept 2 mg	€32,829	€579 (-20%)
Bevacizumab	€29,305	Reference
Faricimab	€29,862	€705 (-2%)
Ranibizumab	€52,201	€84 (-87%)

## Discussion

This study shows that, after three years of treatment, aflibercept 8 mg would be the cheapest treatment option for treatment-naïve AMD patients in the Netherlands. There is a marginal cost difference between aflibercept 8 mg and bevacizumab, which is primarily attributed to the lower medication expenses associated with the latter. The higher medication price of aflibercept 8 mg is offset by its low required injection frequency when compared to the less expensive bevacizumab. Based on the treatment frequency that was observed in clinical trials, aflibercept 8 mg may reduce the injection frequency by 15 injections over a 3-year time horizon, compared to bevacizumab.

Our results are based on list prices, which might be higher than the net price a hospital is paying (i.e., the net price). The break-even analysis shows that aflibercept 2 mg and faricimab are cost-equal to bevacizumab when applying a 33% and 19% discount, respectively. Additionally, our study found that ranibizumab is cost-saving compared to bevacizumab when the medication price is set below €75. Our estimations of the break-even prices hold particular value in light of the upcoming biosimilars in the field as well as the yearly contract negotiations between clinics and healthcare insurers on reimbursement tariffs for anti-VEGF medication [43].

Our study is a follow-up of a previous cost-minimization analysis that compared aflibercept 2 mg, bevacizumab, and ranibizumab in a T&E regimen when the novel anti-VEGFs were not yet available [23]. The studies used a similar set-up, with a 3-year time horizon and cost inputs that were relevant from a healthcare payer’s perspective. While previous analysis found that bevacizumab was the cheapest treatment option based on list prices, current analysis finds that aflibercept 8 mg would be cheaper due to its considerably lower injection frequency. Both cost-minimization analyses are a valuable extension of the budget-impact analysis in the Dutch treatment guideline due to the incorporation of a broader perspective [7]. While the current guideline primarily centres on medication costs and does not differentiate between anti-VEGFs in terms of injection frequency and related impact, the cost-minimization analyses provide a more comprehensive perspective and follow health technology assessment guidelines [7, 24].

The reduced pressure on the healthcare system is especially important given the current substantial strain on ophthalmologic care in the Netherlands. The costs of eye-related disorders amount to approximately one billion euros, with waiting lists of 8.1 weeks in average duration [15, 16]. AMD is one of the most prevalent ophthalmic disorders, and with the ageing of the population, it is expected to become even more prevalent [3, 17]. Moreover, anti-VEGF treatment is time- and resource-intensive, and studies show that patients with nAMD prefer an effective treatment that requires fewer hospital visits [12, 13]. For those reasons, a Dutch T&E report published in 2022 stated that a T&E regimen could reduce over- and undertreatment in patients with nAMD and therefore contribute to reduced pressure on healthcare and patients [17]. The report provided several recommendations on the optimal T&E regimen. For the novel anti-VEGFs, no randomized clinical trials that investigate solely a T&E regimen have been published to date. Therefore, we chose to include the T&E regimen for the established anti-VEGFs and the currently studied SmPC regimens for the recently introduced anti-VEGFs [8]. Similar to the T&E regimen, the flexible regimens of aflibercept 8 mg and faricimab aimed to reduce injection frequency, while preventing undertreatment [22, 27]. Moreover, in the second year, the adjustments in the flexible regimens were similar to those in T&E, with the treatment intervals of aflibercept 8 mg even exceeding 16 weeks, which was the maximal treatment interval in a T&E regimen.

Due to limited data, we made several assumptions that led to uncertainty in our outcomes. First, we employed a 3-year time horizon to estimate the often long-term anti-VEGF treatment, which is in line with Dutch guidelines [24]. As there were no 3-year clinical trial data available, we assumed that the injection frequency in the second and third years was equal. To assess the impact of this assumption, we employed a scenario analysis in which a two-year time horizon was used. In this scenario, bevacizumab led to the lowest treatment costs and was closely followed by aflibercept 8 mg. Considering a 2-year treatment duration, an 2% reduction in the medication price of aflibercept 8 mg would result in equivalent treatment costs to bevacizumab. Additionally, to reduce the impact of our assumption, a break-even analysis was performed, which showed the break-even price per injection frequency over a 3-year time horizon.

Although the assumption of an equal injection frequency in the second and third year appeared realistic according to a trial and some real-world studies [33–37], it introduced uncertainty for the flexible up to 16-week and up-to 24 week regimens. The PULSAR trial demonstrated that, at 96 weeks, approximately 53% of patients who were randomized to the aflibercept 8 mg every 16 weeks arm at baseline had a last-assigned injection interval of ≥ 20 weeks [44], potentially reducing the number of injections in subsequent years. Moreover, although a 3-year time horizon already led to assumptions and uncertainty, it was still an underestimation of the actual treatment duration of nAMD. nAMD is a chronic disease, and the average treatment duration is therefore estimated to exceed this time horizon [45, 46]. As shown in the scenario analysis, the advantage of a reduced injection frequency increases over time, and the relative costs of aflibercept 2 mg, aflibercept 8 mg and faricimab might reduce with a longer time horizon.

A second assumption that we made was that we did not consider variations in disease activity criteria across different clinical trials. While some trials defined disease activity as retinal fluid, others used visual acuity to define disease activity or both. Moreover, while some trials shortened the treatment interval after meeting one of the criteria, others continued with the existing interval after meeting one criterion and only shortened it when multiple criteria were fulfilled. This might have led to discrepancies in the strictness of reinjection and therefore influenced the number of injections in the trials. Nevertheless, the anti-VEGFs showed non-inferiority in terms of clinical vision gains in the included trials, despite differences in treatment regimens [10, 22, 27]. Moreover, the break-even analysis and OWSA showed the impact of a reduced or increased treatment frequency in a detailed manner.

Lastly, another important consideration to make is that there is currently no robust real-world data for aflibercept 8 mg. The focus of the recent novel anti-VEGF agents has been on reducing injection frequency and reaching maximum injection intervals has therefore been a specific goal for the randomized clinical trials [22, 26]. Real-world data would be useful to confirm these favourable injection frequencies.

We used a cost-minimization analysis rather than a cost-effectiveness analysis to compare currently available anti-VEGFs since non-inferiority trials and indirect treatment comparisons demonstrate their clinical non-inferiority concerning vision gain [10, 20–22, 27]. However, for this reason, we omitted the impact of a reduced intravitreal injection frequency on the patient’s well-being. Intravitreal injections accompanied by monitoring visits take time, impacting the daily lives of patients [12, 13]. In a future study, to make a fair comparison, it would be interesting to include the impact of the treatment regimen on the patient and caregivers. In addition, our analysis provides a simplified version of the actual treatment pathway of patients with nAMD and did not include treatment discontinuation or treatment switching. A Dutch real-world study found that a substantial part of nAMD patients switch anti-VEGFs during treatment [37]. The study compares the number of patients that switched in the Netherlands versus a larger international registry and found that in the Netherlands, 58.5% of patients switched from bevacizumab after 3 years. In countries where patients initiate treatment with aflibercept 2 mg or ranibizumab, this rate was notably lower at 28.7%. Switching a treatment might lead to reduced initial efficacy and leads to an extra loading phase, increasing the (economic) impact of treatment. Treatment discontinuation leads to even worse visual acuity outcomes. Including the impact of these real-life treatment pathways might, therefore, contribute to a more accurate representation of the costs associated with anti-VEGF treatment.

Nevertheless, our analysis provides a detailed comparison of the costs associated with different anti-VEGF treatment for nAMD, which is valuable to decision-makers and healthcare providers. The outcomes show that ophthalmologists have achieved considerable healthcare savings by positioning off-label bevacizumab as first-line therapy for many years. However, the recently introduced anti-VEGF therapies seem to provide a solution to increase efficiency, sustainability, and patient satisfaction by reducing the treatment burden for patients, caregivers and clinics of anti-VEGF therapy for nAMD patients in the Netherlands at no additional cost. As our experience of using aflibercept 8 mg in clinical practice grows and the low injection frequency holds true, eventually we should consider adapting the guideline.

In conclusion, our analysis shows that based on the injection frequencies found in clinical trials, aflibercept 8 mg would be the least costly treatment for nAMD in the Netherlands when given for at least three years. This is owing to its lower healthcare costs attributed to its low injection frequency.

## Appendix 1

See Table 8.

**Table 8:** Model assumptions

The treatment effect was omitted due to the non-inferiority concerning vision gain of anti-VEGFs shown in indirect treatment comparisons and clinical trials [10]
Some anti-VEGF medications do not have trials specifically investigating a T&E regimen. In such cases, a flexible regimen is assumed
A T&E regime consists of a loading phase, extension phase and maintenance phase. The loading phase is three monthly injections. In the extension phase treatment intervals can either be extended, maintained, or shortened with 2-week intervals based on disease activity [7]
Following Dutch guideline AMD 2023, for every administration, a control visit is assumed. In the loading/induction phase, two monitoring visits are assumed (i.e., at treatment initiation and the end of the loading phase [7]
serious AEs solely related to intravitreal injections are included. The risk of these adverse events is equal for all anti-VEGFs due to the clinical non-inferiority
As per the Dutch guideline AMD 2023, the diagnosis of nAMD is typically performed using SD-OCT, vision assessment and fundoscopy, and this assessment is repeated at the end of the loading phase. Subsequent monitoring visits or follow-up assessments typically involve the use of SD-OCT alone [7]
The injection frequency is based on (a weighted average of) data collected from available RCTs
The number of injections in RCTs with a duration of 96 weeks is corrected to 104 weeks
It is assumed that the number of injections in the third year is equal to the second year
The base case assumes bevacizumab preparation in a hospital pharmacy

*AE* Adverse event; *RCT* Randomized clinical trials; *SD-OCT* Spectral domain optical coherence tomography; *T&E* Treat-and-extend

## Appendix 2

See Table 9.

**Table 9:** Dutch guidelines regarding T&E regimens

	Dutch treatment guideline							
	Treatment schedule				Treat-and-extend criteria			
Regimen	Loading phase	Minimum and maximum interval	Interval adjustments	Monitoring visits	Vision	Fluid	Haemorrhage	Other
T&E Based on Dutch Guidelines AMD 2023 and Dutch T&E report [7, 17]	Yes (4 doses)	4 weeks to 16 (13) weeks	2-week extension in case of no disease activity If disease activity re-occurs, the advice is to initially return to the interval at which the disease was inactive. In cases of substation disease activity, going back to the loading phase is recommended	At the same visit before every injection assessment with SD-OCT	At diagnosis: best-corrected vision is measured	Every control and diagnosis: active leakage; increase in or existing sub- or retinal fluid assessed by SD-OCT	At diagnosis: Haemorrhage assessed by fundoscopy	At diagnosis: slit lamp examination

*AMD* Age-related macular degeneration; *FAG* Fluorescein angiography; *T&E* Treat-and-extend; *SD-OCT* Spectral domain optical coherence tomography

## Appendix 3

See Table 10.

**Table 10:** Description of included trials

Clinical trial	Phase	Set-up	Duration	Drug(s) and regime(s)	Primary endpoint(s)	Source
TREX	Phase IIIb	Randomized, controlled, multi-centre study	24 months	Ranibizumab: monthly, T&E	Mean BCVA change from baseline (ETDRS letters)	[28]
LUCAS	Phase IIIb	Randomized, double-masked, multi-centre study	24 months	Ranibizumab and bevacizumab: T&E	Mean BCVA change as measured on the ETDRS chart	[18]
TREND	Phase IIIb	Randomized, assessor-masked, multi-centre study	12 months	Ranibizumab: T&E regime and monthly regime	Change in BCVA from baseline to the end of the study (ETDRS letters)	[29]
InEye	Phase IV	Randomized, open-label, multi-centre study	12-months	Ranibizumab: T&E, bimonthly and Pro re nata	Mean change in BCVA from baseline over a 12-month period	[32]
CANTREAT	Phase IV	Randomized, open-label, multi-center, noninventory, post-authorization study	24 months	Ranibizumab: Monthly, T&E	The effectiveness of 2 treatment regimens by assessing the mean change in BCVA (ETDRS letters) from baseline to month 24	[31]
ALTAIR	Phase IV	Randomized, open-label, multi-centre study	96 weeks	Aflibercept 2 mg: T&E regimes with 2-week or 4-week interval adjustment	Change in BCVA letters from baseline	[20]
ARIES	Phase IIIB/IV	Randomized, open-label, multi-centre study	24 months	Aflibercept 2 mg: T&E after loading/induction dose and T&E in the second year	Change in the BCVA (ETDRS letters) from randomization (W16) to W104	[30]
TENAYA/LUCERNE	Phase III	Randomized, double-masked, multi-centre study	96/104? weeks*	Faricimab: flexible Q8/Q12/Q16 regimen Aflibercept 2 mg: fixed 8-week injection interval regimen	Mean change in BCVA (ETDRS letters) from baseline averaged over weeks 40, 44, and 48	[26, 27]
PULSAR	Phase III	Randomized, double-masked, multi-centre study	96 weeks*	Aflibercept 8 mg: flexible 12- and 16-week injection interval regimen arm, with extension to max. 24-week injection interval regimen from year 1 Aflibercept 2 mg: fixed 8-week injection interval regimen year 1 and 2	mean change in BCVA as measured by ETDRS letter score at week 48	[21, 22]

*BCVA* Best-corrected visual acuity; *ETDRS* Early Treatment Diabetic Retinopathy Study; *T&E* Treat-and-extend

## Appendix 4

See Fig. 3.
